# 12-hydroxyeicosatetraenoic acid is associated with variability in aspirin-induced platelet inhibition

**DOI:** 10.1186/s12950-014-0033-4

**Published:** 2014-10-23

**Authors:** Benjamin H Maskrey, Gordon F Rushworth, Matthew H Law, Andrew T Treweeke, Jun Wei, Stephen J Leslie, Ian L Megson, Phillip D Whitfield

**Affiliations:** Department of Diabetes and Cardiovascular Science, University of the Highlands and Islands, Old Perth Road, Inverness, IV2 3JH UK; Highland Clinical Research Facility, Inverness, UK; Cardiac Unit, Raigmore Hospital, Inverness, UK

**Keywords:** Aspirin, Platelet, 12-HETE, Thromboxane, Eicosanoids, Anti-inflammatory

## Abstract

**Background:**

Aspirin is one of the most widely used non-steroidal anti-inflammatory drugs (NSAIDs). It is also a commonly used anti-platelet drug, which inhibits the formation of the platelet activator, thromboxane A_2_ (TxA_2_) via inhibition of cyclooxygenase-1 (COX-1). However, the presence of a patient subset that fails to respond to aspirin despite reduced TxA_2_ concentrations suggests that the effect of aspirin might be more complex than exclusive COX-1 inhibition.

**Methods:**

In this study we evaluated the impact of *in vivo* oral administration of a standard anti-platelet dose (75 mg) of aspirin in healthy volunteers on the acute impact of *in vitro* collagen-mediated platelet aggregation and generation of platelet-derived TxA_2_ and the 12-lipoxygenase (LOX) metabolite 12-hydroxyeicosatetraenoic acid (12-HETE). The eicosanoids were quantified using liquid chromatography-tandem mass spectrometry (LC-MS/MS).

**Results:**

Low-dose aspirin administration not only inhibited TxA_2_ generation but also decreased the production of 12-HETE. Furthermore, a significant correlation was observed between the levels of 12-HETE and collagen-induced platelet aggregation. Pre-treatment of platelets with the 12-LOX inhibitor, baicalein, prior to activation attenuated platelet aggregation.

**Conclusions:**

These findings support a role for 12-HETE as a pro-aggregatory eicosanoid in platelet function and suggest a role for 12-HETE in variable sensitivity to aspirin. The study also highlights a potentially important mechanism by which aspirin impacts upon eicosanoid generation.

## Background

Aspirin (acetylsalicylic acid) is one of the most widely used non-steroidal anti-inflammatory drugs (NSAIDs). In addition to possessing anti-inflammatory properties at high doses (1 g/day) [1] aspirin also demonstrates effective cardioprotective properties at lower doses (75–150 mg/day) [2]. The proposed mechanism by which aspirin acts is by irreversible inhibition of cyclooxygenase 1 (COX-1) [3]. Enzyme acetylation effectively blocks formation of the prostaglandin intermediates, PGG_2_ and PGH_2_ and their resultant downstream bioactive products [4]. The cardioprotective anti-platelet property of low-dose aspirin is attributed to irreversible inhibition of synthesis of a pro-thrombotic COX-1-derived eicosanoid, thromboxane A_2_ (TxA_2_) [5], which is derived from the polyunsaturated fatty acid substrate, arachidonic acid (AA).

Platelet specificity of aspirin action is believed to stem from the fact that platelets lack nuclear DNA, rendering them incapable of *de novo* biosynthesis of COX-1, ensuring that inhibition of COX-1 prevails for the lifespan of the platelet. There is an increasing awareness of clinical variability in anti-thrombotic response to aspirin, with a significant proportion of patients failing to respond, a phenomenon referred to as ‘aspirin resistance’ [6]. Estimates of aspirin resistance vary widely (5-45%) [7], but resistant patients are known to display an increased risk of cardiovascular morbidity [8]. There is, however, a lack of association between aspirin-mediated inhibition of platelet function and inhibition of TxA_2_ synthesis. This apparent anomaly has been attributed to a perceived lack of reliability of platelet function assays, on the assumption that reduced inhibition of TxA_2_ is the main contributory factor in aspirin resistance [9].

Whilst TxA_2_ is undoubtedly an important mediator in the platelet activation process, to date little attention has been paid to other eicosanoids generated by platelets. 12-hydroxyeicosatetraenoic acid (12-HETE), formed by the enzyme 12-lipoxygenase (12-LOX), is another major platelet-derived eicosanoid, although its role in platelet function remains unclear. Reports have suggested 12-HETE to have both anti- and pro-thrombotic properties [10-12]. It has also been suggested to play a role in promoting expression of platelet CD62 (P-selectin) [13] and is elevated in patients with essential hypertension [14]. The increasing recognition of the potential importance of 12-HETE in platelet function has led to a recent resurgence of research in the area, which has resulted in the development of novel 12-LOX inhibitors to help elucidate the function of platelet 12-HETE and its potential as a novel anti-platelet target [15,16]. The impact of aspirin on 12-HETE is not yet fully understood. In this study we have investigated the association of TxA_2_ (measured as its degradation product TxB_2_) and 12-HETE levels, with low-dose aspirin-mediated inhibition of platelet aggregation in healthy volunteers.

## Methods

### Subjects

Healthy volunteers (*n* = 19) were recruited through the Highland Clinical Research Facility. Subjects were free from all non-steroidal anti-inflammatory drugs (NSAIDs) two weeks prior to blood collection. A 5 ml blood sample was drawn into trisodium citrate tubes (Monovette®, Sarstedt, Leicester, UK) from the antecubital fossa (21G) before and 1.5 h after oral dosing with aspirin (Actavis, Barnstaple, UK; 75 mg). For inhibitor studies, a further three healthy volunteers were recruited and blood drawn as above. The study was approved by the North of Scotland Research Ethics Committee and the University of the Highlands and Islands Ethics Committee, and was in accordance with the Declaration of Helsinki and its amendments, with all volunteers providing written informed consent.

### Measurement of platelet aggregation and inhibitor studies

Platelet aggregation was measured in response to collagen in platelet-rich plasma (PRP) derived from the freshly drawn blood samples immediately before and 1.5 h after aspirin administration. Briefly, fresh blood was centrifuged (120 *g* for 10 min at room temperature), PRP aspirated and the platelet count obtained on an AcT Diff 8 Blood Counter (Beckman Coulter, High Wycombe, UK). The remaining blood sample was centrifuged (1000 *g* for 10 min at room temperature) and the platelet poor plasma (PPP) aspirated for use as a reference and to dilute the PRP to a standard platelet count (150 × 10^6^/ml). Standardized PRP samples were aliquoted (500 μl) into glass cuvettes with a micro-stirrer and pre-warmed to 37°C prior to addition of type I collagen (Labmedics, Manchester, UK; 2.5 μg/ml). Aggregation was recorded for 6 min using a Chrono-Log 700 platelet aggregometer (Chrono-Log, Haverford, PA, USA), at which point the activated platelets were immediately stored at −80°C for subsequent eicosanoid analysis. Aggregation was quantified as the area under the curve (AUC) over the 6 min period post-activation. To determine the effect of 12-LOX inhibition on platelet aggregation, platelets were pre-incubated for 10 min with either 100 μM baicalein (Tocris Bioscience, Bristol, UK) or DMSO as a vehicle control at a final concentration of 0.5% (v/v) prior to addition of 1.25 μg/ml collagen; aggregation was measured as described above and samples retained at the end of the aggregation assay for subsequent eicosanoid measurements.

### LC-MS/MS analysis of platelet eicosanoids

Eicosanoids were isolated from the PRP following collagen activation using solid-phase extraction (SPE) chromatography (C18 500 mg, 6 ml, Waters, Manchester, UK). 1 ml ice-cold methanol containing 1 ng of the deuterated internal standards, PGE_2_-d_4_ and15-HETE-d_8_ (Cayman Chemical, Ann Arbor, MI, USA), was added to 0.5 ml PRP to precipitate proteins, and following centrifugation (600 *g* for 10 min at 4°C), the supernatant was removed, diluted to <10% methanol content by the addition of 9 ml ddH_2_O and acidified to pH 3.5 with 1 M HCl. Acidified samples were immediately added to SPE cartridges, which had been conditioned with 2 × 6 ml methanol, followed by 2 × 6 ml ddH_2_O. Samples were then washed with a further 6 ml ddH_2_O and 2 × 5 ml hexane before elution of the eicosanoids with 2 × 3 ml ethyl acetate. The ethyl acetate fraction was dried under vacuum and then resuspended in 100 μl 50:50 (v/v) solvent A:solvent B where solvent A consisted of H_2_O:methanol 90:10 (v/v) containing 0.1% (v/v) acetic acid and solvent B consisted of methanol containing 0.1% (v/v) acetic acid. All solvents were LC-MS grade (Fisher Scientific, Loughborough, UK).

Eicosanoids were separated on a Kinetex 1.7 μm XB-C18 column (100 × 2.1 mm) (Phenomenex, Macclesfield, UK) column using a Thermo Accela 1250 UHPLC system (Thermo Scientific, Hemel Hempstead, UK). The gradient was 45-100% solvent B over 18 min at a flow rate of 400 μl/min. The LC effluent was directed into the source of a Thermo TSQ Quantum Ultra triple quadrupole mass spectrometer. The instrument was operated in negative ion mode and data were acquired using the selected reaction monitoring (SRM) mode. Eicosanoids were identified on the basis of their characteristic ion pairs (TxB_2_, 369/169; PGE_2_-d_4_, 355/193; PGE_2_, 351/189; 12-HETE, 319/179; 15-HETE-d_8_, 327/226) and matching retention time with authentic standards. Data were acquired and analysed using Thermo LCquan v2.6 software. Concentrations of TxB_2_ and 12-HETE were determined by comparison to a calibration curve run in parallel for each compound and adjusted for recovery by reference to amounts of the PGE_2_-d_4_ and 15-HETE-d_8_ internal standards respectively.

### Statistical analysis

Data were compared with two-tailed, paired Student’s *t*-test or analysed using regression analysis (Pearson’s). A *P*-value of <0.05 was considered statistically significant in all cases with data expressed as mean ± SEM. The statistical analysis was performed using GraphPad Prism v5.0 software.

## Results

### Effect of aspirin on platelet eicosanoid levels

**Figure 1:**
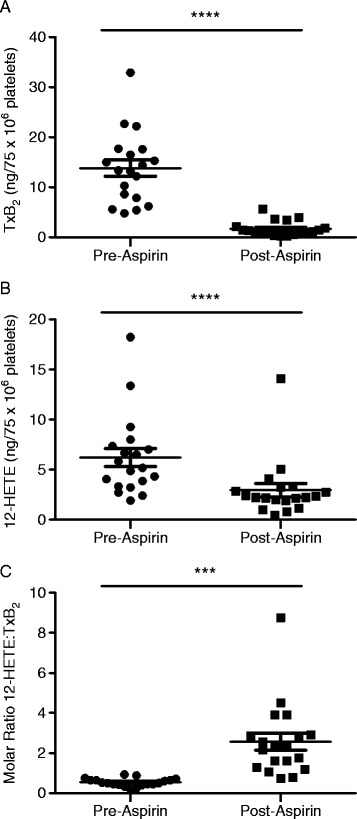
**Low-dose aspirin decreases levels of TxB**_**2**_ **and 12-HETE.** Concentrations of **(A)** TxB_2_ and **(B)** 12-HETE were reduced in collagen-activated platelets following 75 mg aspirin oral administration to healthy volunteers (*n* = 19). **(C)** 12-HETE:TxB_2_ molar ratio is elevated following aspirin-treatment. Data are expressed as mean ± SEM. ****P < 0.0001 and ***P < 0.0005.

One of the main eicosanoids detected from collagen-stimulated platelets was TxB_2,_ the stable isomer of TxA_2_ and a surrogate measure of TxA_2_ generation. Prior to aspirin treatment there was a wide distribution of TxB_2_ concentrations in the study cohort with a mean value of 13.8 ± 1.6 ng/75 × 10^6^ platelets (mean ± SEM). In keeping with the recognised concept of aspirin activity as a COX-1 inhibitor, oral administration of low-dose aspirin led to a statistically significant decrease in platelet TxB_2_ concentrations in all of the subjects, resulting in a mean TxB_2_ concentration of 1.7 ± 0.3 ng/75 × 10^6^ platelets (Figure 1A).

Interestingly, aspirin also induced a highly statistically significant (*P* < 0.0001) decrease in platelet 12-HETE generation, reducing levels prior to stimulation from a mean of 6.2 ng to 2.9 ± 0.7 ng/75 × 10^6^ platelets (Figure 1B). This suggests that, in addition to inhibition of COX-1, aspirin also impacts upon the 12-LOX pathway to decrease 12-HETE levels. In contrast to TxB_2_, 12-HETE inhibition was less pronounced and more variable among subjects (mean inhibition =53 ± 20%; range, 20-84%), whereas TxB_2_ inhibition was strong and consistent between subjects (mean inhibition =88 ± 7%; range, 78-95%). Whilst considerable inter-subject variation in both TxB_2_ and 12-HETE levels pre-aspirin treatment was observed, when the relative amounts of TxB_2_ and 12-HETE were compared, the 12-HETE:TxB_2_ molar ratio was found to be similar in all subjects, regardless of the initial eicosanoid concentration (Figure 1C). However, post-aspirin treatment, this ratio was considerably higher, a result that indicates an excess of 12-HETE compared to TxB_2_ in the platelets. Concentrations of PGE_2_ were lower than TxB_2_ and 12-HETE, with a mean value of 0.46 ± 0.06 ng/75 × 10^6^ platelets. Following aspirin administration PGE_2_ was undetectable in the majority of samples (data not shown).

### Aspirin and platelet aggregation

**Figure 2:**
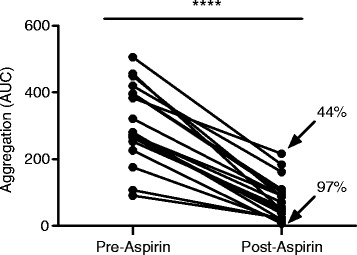
**Platelet aggregation is decreased by low-dose aspirin.** Collagen-induced platelet aggregation is significantly decreased following oral administration of 75 mg aspirin to healthy volunteers (*n* = 19). Data are expressed as mean ± SEM. ****P < 0.0001.

Considering the functional response of platelets to aspirin treatment, low-dose aspirin treatment significantly (*P* < 0.0001) decreased the platelet aggregatory response to collagen in all subjects (Figure 2). The greatest observed aspirin-mediated reduction in platelet aggregation was 97%, whilst the lowest reduction in aggregation caused by aspirin resulted in only a 47% decrease in the aggregatory response.

### Correlations between eicosanoid levels and platelet aggregation

**Figure 3:**
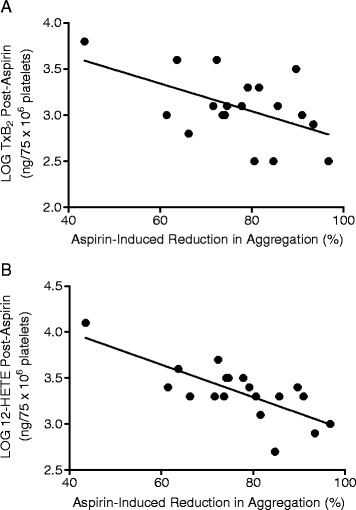
**Correlations of TxB**_**2**_ **and 12-HETE and levels with platelet aggregation**. Post-aspirin concentrations of **(A)** TxB_2_ and **(B)** 12-HETE correlate with aspirin-induced reduction in platelet aggregation. On exclusion of the point with markedly lower inhibition, the correlation between 12-HETE levels and the platelet aggregatory response remains statistically significant (*P* < 0.02).

When eicosanoid levels generated from platelets post-aspirin treatment were compared with the aspirin-induced reduction in aggregation, a statistically significant correlation (*P* = 0.02; Pearson correlation coefficient of *r* = 0.52) was observed between TxB_2_ levels and aspirin-mediated decrease in platelet aggregation (Figure 3A). Elevated TxB_2_ levels were associated with smaller reductions in aggregation, whilst the lowest levels of TxB_2_ correlated with the greatest percentage decrease in aggregation. However, as shown in Figure 3B, an even stronger relationship was found to exist between post-aspirin 12-HETE levels and the platelet aggregatory response, which was statistically significant (*P* = 0.0003) and produced a Pearson correlation coefficient of *r* =0.74. Similar to TxB_2_, the lowest levels of 12-HETE correlated with the greatest aspirin-induced reduction in aggregation.

### 12-LOX inhibition and platelet aggregation

**Figure 4:**
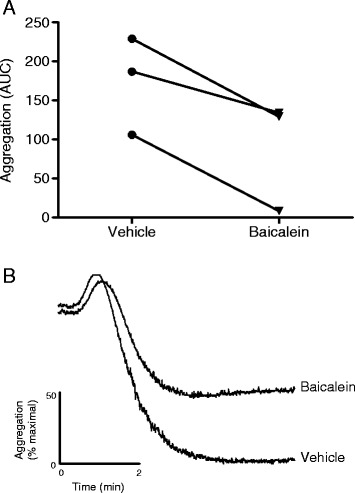
**Inhibition of 12-LOX reduces platelet aggregation.** Platelets were pre-treated with or without 100 μM baicalein prior to aggregation with 1.25 μg/ml collagen. Platelet aggregation was measured for 6 min and data expressed as **(A)** area under the curve (AUC). (*n* = 3, mean ± SEM). Panel **B** shows a representative aggregometry trace.

To investigate a functional role for 12-HETE in platelet aggregation, 12-LOX was inhibited using baicalein. As shown in Figure 4A, treatment with 100 μM baicalein significantly reduced (*P* = 0.032) platelet aggregation in response to collagen activation (mean reduction in aggregation =55 ± 11%). Figure 4B shows representative aggregometry traces. To confirm effective baicalein-induced inhibition of 12-LOX, levels of 12-HETE were measured by LC-MS/MS and revealed a mean reduction in 12-HETE to 52 ± 6% with baicalein treatment.

## Discussion

These findings demonstrate that, as well as inhibiting TxA_2_ generation through COX-1, aspirin additionally impacts upon 12-LOX-mediated synthesis of 12-HETE. Whilst the effect of aspirin on TxA_2_ was universal and comprehensive, indicating effective aspirin-mediated COX-1 inhibition, the variation in efficacy on 12-HETE inhibition was also closely associated with the extent of inhibition of aggregation. Indeed, the subject with the highest levels of 12-HETE was found to have the lowest aspirin-induced reduction in platelet aggregation. This suggests that aspirin-mediated inhibition of 12-HETE generation may be of use in predicting the efficacy of aspirin with regard to platelet function. Whilst generally considered to be a classic COX-1 inhibitor, aspirin has previously been shown to impact upon the platelet 12-LOX pathway, and can reversibly inhibit 12-HETE generation in a dose-dependent manner [17,18]. This effect appears to be unique to aspirin rather than being due to secondary effects caused by COX inhibition as treatment with indomethacin (COX-1 inhibitor) has no impact upon 12-HETE levels [19].

Considerable heterogeneity of platelet response to aspirin was observed between the healthy volunteers in this study, despite effective TxB_2_ inhibition. This observation parallels a recent study which showed that aspirin reduced TxB_2_ levels in all subjects tested [20] further supporting the notion that an alternative mechanism to TxB_2_ may be involved in variable sensitivity to aspirin. Our study examined the acute (1.5 hours) effect of aspirin treatment on eicosanoid levels and platelet function. Despite aspirin being an irreversible inhibitor of platelet COX-1, it is recognised that cumulative dosing of aspirin may be required to achieve maximal inhibition of TxB_2_ generation, although how cumulative dosing effects 12-HETE inhibition are currently unknown [21].

12-HETE has been suggested to play a role in platelet function and is thought to contribute to both platelet adhesion and aggregation [19,22] although its exact role is unclear. Addition of 12-HpETE, the hydroperoxy precursor of 12-HETE has been demonstrated to play a ‘priming’ role and increase aggregation of washed platelets [23,24]. However, other studies have shown that addition of exogenous 12-HpETE inhibits both collagen and thrombin-induced platelet aggregation [25,26] suggestive of anti-aggregatory properties.

Pharmacological inhibition of 12-LOX diminishes aggregation of washed platelets, further suggesting a role for 12-HETE in promoting platelet aggregation [11,27,28]. Other evidence for a pro-aggregatory role of 12-HETE comes from a recent study that showed a significant decrease in aggregation of washed platelets in response to collagen with a newly developed inhibitor, NCTT-956 that is highly specific for 12-LOX [29]. These results are consistent with our experimental findings using baicalein, although it should be noted that this inhibitor may have off-target effects on other eicosanoid pathways. The functional importance of platelet 12-LOX is also emphasized by the fact that 12-LOX-deficient mice display elevated mortality in an ADP-induced model of thromboembolism [30] and inhibition of 12-LOX inhibit coronary thrombosis in a canine model [31]. Furthermore, in patients receiving heparin treatment, platelet-derived 12-HETE has been shown to be elevated, whilst TxB_2_ levels were unchanged [32]. This was suggested as a possible mechanism to explain the phenomenon of transient heparin-induced platelet activation.

The signalling pathways involved in eicosanoid-dependent platelet activation are complex and not yet fully understood. Collagen is known to stimulate platelet 12-LOX activity via the glycoprotein VI receptor, in a PECAM-1 and PKC-dependent pathway [33]. Release of AA from membrane phospholipids by PLA_2_ activation is a critical step in eicosanoid biosynthesis and a recent study has shown that COX-1 and 12-LOX are linked to different PLA_2_ subsets, suggesting that TxA_2_ and 12-HETE are generated from different intracellular pools of AA [34]. 12-HpETE is known to modulate platelet COX activity, suggesting a degree of ‘cross-talk’ between the LOX/COX pathways although the exact mechanism is unclear.

Some studies have reported a stimulatory effect of low concentrations of 12-HpETE on COX-1 activity [30], whilst others have shown that 12-HpETE inhibits COX-1 by altering calcium homeostasis via activation of soluble guanylate cyclase [26]. The exact nature of the inhibitory mechanism of aspirin on the 12-LOX pathway is unclear at present. However, it has been suggested that aspirin exerts its impact by inhibiting the glutathione peroxidase-mediated conversion of 12-HpETE to 12-HETE, preventing 12-HETE formation [35]. COX-1 has a bifunctional activity producing the labile peroxide, PGG_2_, which is reduced to the alcohol PGH_2_ via an endoperoxidase mechanism. PGH_2_ is then further metabolised by various prostanoid-generating enzymes. This bears a striking similarity to the mechanism of 12-HETE generation, whereby 12-LOX catalyses the regio- and stereo-specific incorporation of molecular oxygen to form the hydroperoxide (12-HpETE), which is then reduced to the corresponding alcohol (12-HETE). Therefore, it is possible that aspirin may be acting in a similar manner, and preventing the reduction of 12-HpETE.

Aspirin remains, and is likely to remain for some time, the mainstay of anti-platelet therapy in vascular patients and so a full understanding of its mechanisms of action is essential. Here we have shown for the first time a correlation between aspirin-mediated platelet aggregation and 12-HETE generation. This finding highlights the importance of the platelet 12-LOX pathway as an additional mechanism by which aspirin can modulate the complex network of AA metabolism, with potential effects on thrombotic events. This study also underlines the importance of monitoring interconnecting pathways when investigating the inhibitory mechanism of pharmacological agents. In future work the investigation of the mechanism and nature of aspirin inhibition of 12-LOX could be extended by using platelet markers of activation *ex vivo* e.g. P-selectin, larger sample sets and disease cohorts such as patients with type 2 diabetes or cardiovascular disease.

## Conclusions

This study draws attention to the potential importance of the 12-LOX pathway in platelet function, specifically aspirin-mediated platelet inhibition. The findings suggest that the variability of platelet function in response to aspirin is linked to the inhibition of 12-HETE generation, which may be acting in concert with TxA_2_ inhibition. As such, the work may have implications for our understanding into how aspirin impacts upon eicosanoid generation.

## Abbreviations

AA: Arachidonic acid
COX: Cyclooxygenase
HETE: Hydroxyeicosatetraenoic acid
HpETE: Hydroperoxyeicosatetraenoic acid
LOX: Lipoxygenase
NSAID: Nonsteroidal anti-inflammatory drug
PG: Prostaglandin
PRP: Platelet-rich plasma
Tx: Thromboxane
